# Comparative effects of fractional radiofrequency and microneedling on the genitalia of postmenopausal women: Histological and clinical changes

**DOI:** 10.1016/j.clinsp.2022.100117

**Published:** 2022-09-29

**Authors:** Rafaella Rêgo Maia, Ayane Cristine Sarmento, Rodrigo Marcel Valentim da Silva, Eneida de Morais Carreiro, Stephany Luanna Queiroga Farias, Ciro Dantas Soares, Patrícia Froes Meyer, Ana Katherine Gonçalves

**Affiliations:** a Health Sciences Postgraduate Program, Universidade Federal do Rio Grande do Norte (UFRN), Natal, RN, Brazil; b Physiotherapy Department, Centro Universitário do Rio Grande do Norte (UNI-RN), Natal, RN, Brazil; c Physiotherapy Department, Universidade Estácio de Sá, Natal, RN, Brazil; d Physiotherapy Department, Universidade Potiguar (UnP), Natal, RN, Brazil; e Tomatopathology Department, Universidade Estadual de Campinas (UNICAMP), Campinas, SP, Brazil; f Department of obstetrics and gynaecology, Universidade Federal do Rio Grande do Norte (UFRN), Natal, RN, Brazil

**Keywords:** Menopause, Flaccidity, Radiofrequency, Microneedling

## Abstract

**Objectives:**

The authors aimed to evaluate clinical and histological changes induced by Fractional Radiofrequency (FRF) and microneedling in vulvar tissue.

**Methods:**

Thirty postmenopausal women were randomly divided into G1 (FRF) and G2 (microneedling) groups. Sub-ablative FRF was executed using disposable fractionated electrodes with an intensity of 8 mJ. Microneedling was performed using a derma roller system. The authors evaluated before and after treatment using the Vaginal Laxity Questionnaire (VLQ), EuroQol Five-Dimensional (EQ-5D) questionnaire, and the Blatt and Kupperman Menopausal Index (BKMI). Additionally, the authors performed biopsies of the labia majora for histological analysis pre- and post-treatment. Data were expressed as mean (± standard deviation). A paired *t*-test was used for intra-group comparison (pre- and post-treatment), with an independent *t*-test used to compare intergroup data (both pre- and post-treatment).

**Results:**

In the G1 group, the VLQ values showed differences compared to the pre-treatment values with the data obtained 60 days after the beginning of the sessions (p = 0.01). Similarly, the data changes of the G2 group proved to be significant (p = 0.001) across the same time interval. In comparing the groups, VLQ values were not different (p > 0.05). Regarding histological analysis, FRF demonstrated improvement concerning the number of fibroblasts, blood vessels, and fatty degeneration (p < 0.05) compared to the control. Additionally, FRF and microneedling samples showed higher type III collagen and vimentin expression in the immunohistochemical analysis (p < 0.05).

**Conclusions:**

The therapies were found to be effective in treating the flaccidity of the female external genitalia. Additionally, histological changes were observed after interventions suggesting collagen remodeling.

## Highlights

• Radiofrequency is effective in flaccidity.
• Radiofrequency provokes collagen remodeling.

## Introduction

In menopause, there is a marked reduction in estrogen levels, as a result of which the authors have different changes in the skin. The first perceived change is an increased dryness, followed by a decrease in firmness and elasticity, corresponding to decreased sebum production, collagen content, dermal thickness, and elastin fibers.^1^^,^^2^ During this period, dermal collagen is reduced by approximately 2.1% per year, with skin thickness decreasing by 1.1% over the same period. Studies have found that, in the first five years after menopause, the resulting loss of this protein can reach up to 30%.^3^^,^^4^

Additionally, within the female genitals, these changes have various consequences. The latter include vaginal dryness, pruritus, dyspareunia, discomfort, burning, dysuria, and genitourinary infection. Therefore, menopause directly impacts women's health and overall quality of life, with direct consequences occurring within their most intimate region, especially concerning an increased sagging of their external genitalia.^5^, ^6^, ^7^

Several therapeutic strategies have been proposed to reverse or minimize these changes, including systemic and topical treatments, surgical procedures, and, recently, non-invasive procedures, to improve the appearance of the skin in the genital region and increase your self-esteem and sexual satisfaction. However, due to the risks and high costs of surgical procedures, and the contraindications associated with hormone replacement therapy, new non-invasive therapeutic options are needed to treat these changes.^8^^,^^9^

New treatment modalities include laser therapies ‒ such as fractional CO_2_ or erbium (Er: YAG) ‒ as well as Radio Frequency (RF) devices and microneedling,^10^, ^11^, ^12^, ^13^ which have been developed to improve general skin and mucosal tropism. These new therapeutic resources stimulate fibroblasts, and promote neocolagenesis and neoelastogenesis, through multiple micropunctures, which generate an inflammatory process that stimulates the production of new cells.^14^^,^^15^ Previous studies have reported the efficacy of fractional radiofrequency on female genitalia, both for vulvar flaccidity and functionality, in vaginal lubrication, and increased concentration of type III collagen. In addition, its benefits are associated with a low incidence of adverse events, most are considered light, and a high rate of rapid and positive results.^13^^,^^16^, ^17^, ^18^

Usually, the efficacy of RF devices for genitourinary conditions is mainly based on subjective patient-reported outcomes. However, limited studies have evaluated pre- and post-RF treatment histological changes. Thus, this study aims to evaluate clinical and histological changes induced by Fractional Radiofrequency (FRF) and microneedling in vulvar tissue.

## Material and methods

This pilot clinical trial was conducted at the gynecological unit of a university hospital between 2021‒2022. All the participants provided written informed consent. The allocation sequence was based on a list generated by the Software Research Randomizer™, and volunteers were allocated according to the sequence they were evaluated.

The sample was calculated using OpenEpi based on the study by Caruth,^19^ which presented the primary outcome for the treatment of vulvar sagging. A confidence interval of 95% and study power of 80% were adopted; thus, it was necessary to include 15 patients per group, totaling a sample of 30 volunteers.

The authors included sedentary women of up to five years post-menopause, aged between 50 and 60, with flaccidity in their external genitalia, and giving birth at least once. All potential participants who had used hormone replacement therapy experienced changes in their sensitivity, and had active sexually transmitted diseases at the time of this study, with pacemakers, collagen diseases, and dermatological conditions were all excluded.

### Treatment protocol

Treatment with sub-ablative FRF was performed using the Tonederm™ device (RS, Brazil). Its application was performed using disposable fractionated electrodes containing 25 needles of 1 mm length and an intensity of 8 mJ. Microneedling was performed using a derma roller system with 192 needles, as manufactured by the Doutor da Estética™ (São Paulo, Brazil). The roller's movement was directed in horizontal, vertical, and oblique positions until hyperemia was reached. The procedure lasted for approximately 40 min. The area of application for these procedures was the labia majora. Initially, a cream anesthetic (10% lidocaine) was applied to all volunteers 20 min prior to them undergoing this procedure. Each group underwent two sessions per month. The authors justified the period once after the treatment, tropoelastin, responsible for the elasticity and procollagen 1 and 3, which change into collagen, remained stimulated for 28 days. Thus, the reassessments occurred in two stages: 30 and 60 days after the initial session.^20^

### Evaluation procedures

The volunteers were instructed to complete the Global Aesthetic Improvement Scale (GAIS) at the end of the treatment period. Initially, the volunteers were instructed to answer the questionnaire without looking at any comparative photos of their genitalia from before and after treatment. Then, during the second phase, they received these images for a self-analysis of the results and then proceeded to answer the questionnaire again.

A physical therapy questionnaire was administered to collect both clinical and sociodemographic data. Vulvar flaccidity was assessed using the Vaginal Laxity Questionnaire (VLQ). The VLQ's response options are ordered across seven levels for vaginal laxity/tension.^21^ The categorical response levels were then translated into ordinal scores for statistical analysis (the response options include ‘Very loose’ = 1, ‘Moderately loose’ = 2, ‘Slightly loose’ = 3, ‘Neither loose nor tight’ = 4, ‘Slightly tight’ = 5, ‘Moderately tight’ = 6, and ‘Very tight’ = 7).

Another condition assessed in this study was the participants’ quality of life through using the EuroQol Five-Dimensional (EQ-5D) questionnaire that the authors adapted to the theme of female sexuality. The EQ-5D in this study contained five questions, which were scored as follows: excellent (21 to 25 points), good (16 to 20 points), moderate (11 to 15 points), bad (6 to 10 points), and poor (5 points).^22^ Furthermore, the authors utilized the Blatt and Kupperman Menopausal Index (BKMI) to assess the participants’ symptoms, with the scores being categorized as follows: mild (up to 19 points), moderate (from 20 to 35), and severe (above 35).^23^ At the end of the treatment period, the volunteers were asked to answer a questionnaire on their satisfaction with the treatment classification based on the GAIS.^24^

A blinded physician evaluated the photographs to assess each treatment's efficacy and its possible side effects. The photographic records were taken via a semi-professional camera (Canon™, SX530 HS, Japan). The volunteers were placed supine, with abducted and flexed lower limbs, with only their naked genital region being presented. The distance between their feet and knees was 30 cm, with the camera being positioned 20 cm away from their genital region and fixed on a tripod. The photographs were sent to six experienced evaluators in gynecology, dermatology, and urogynecological physiotherapy to assess the changes in the participants’ conditions.

### Histological analysis

Tissue specimens were obtained through a skin biopsy, using the “Punch” technique performed by a gynecologist. The authors performed a sample withdrawal in each group 30 days after the application of the second therapy session and histological and immunohistochemical analysis was performed.

Specimens of control healthy and treated skin samples of the adjacent area were obtained using 2-mm biopsy punches 60 days after treatment. Each sample was immediately placed in 10% neutral buffered formalin and processed for histotechnical analyses as described previously. Sections (5 μm) were cut, placed onto slides, stained with hematoxylin and eosin, and with Picrosirius Red (for 1 h). An attached camera showed the stained tissues under a binocular light microscope (Olympus CX31, Hamburg, Germany). Photomicrographs of various microscopic fields were taken at different magnification levels (× 40, × 100, or × 400), and the collagen content was measured with a Software Leica Application Suite, version 2.8.1 (Leica Microsystems GmbH, Wetzlar, Germany).

### Immunohistochemical assay

For the immunohistochemical assay, sections (3 μm) were cut slides, placed onto syalinized slides, and then incubated (expression of vimentin, cellularity of the connective tissue, and focal expression of type III collagen). The slides were washed and incubated with secondary Horseradish Peroxidase (HRP)-conjugated antibody using the Mouse/Rabbit ImmunoDetector DAB HRP Brown Detection System (BioSB®, Santa Barbara, CA, USA) and revealed with 3.3 Diaminobenzidine-hydrochloride (DAB) producing a brown precipitate. Slides were then counterstained with hematoxylin. The Image J < UNK > software manually counted inflammatory cells positive for the antibodies were manually counted in the Image J® software. Scores were created based on the percentage of positive cells, ranging from (0.1 to 4).

### Statistical analysis

Data analyses were performed using the Statistical Package for the Social Sciences (SPSS) for Windows version 24.0, with a significance level of p ≤ 0.05. Data are expressed as mean (± standard deviation). A paired *t*-test was used for intra-group comparison (for both pre- and post-treatment), with an independent *t*-test being used to compare intergroup data (both pre- and post-treatment).

### Ethics

All study participants provided informed consent to participate in this research. The study was conducted according to the ethical standards outlined in the Declaration of Helsinki (1983), with the local division ethics committee approving this protocol (n°  21336819.9.0000.5537) and it then being registered in the Brazilian Clinical Trials Registry (ReBec): n° RBR- 32jnbm – n° U1111-1242-8225.

## Results

**Fig. 1:**
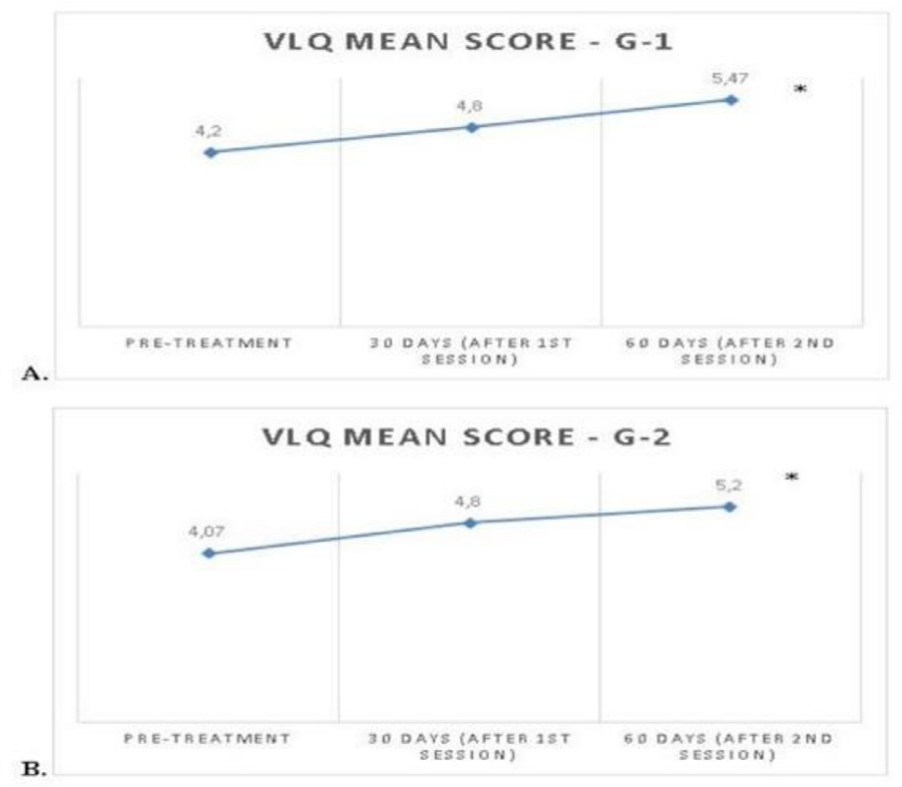
Mean Vaginal Laxity Questionnaire (VLQ) scores from the pretreatment assessment, after 30 days’ post-treatment, and then after 60 days’ post-treatment: (A) G1 fractional radiofrequency; (B) G2 Microneedling. ^a^ Significant results at p≤0.05 for the comparison between the pre-treatment and after 2nd session at the 60-day mark.

The study included 30 participants, Table 1 illustrates their demographic and other characteristics relevant to the investigations of the two study groups. Compared to the base assessment, the VLQ mean scores showed that all the participants’ subjective perceptions about their vaginal tightness had improved significantly by at least one level. The VLQ values for the G1 group showed significant differences when comparing the pre-treatment values with the data obtained 60 days after the beginning of the sessions (p = 0.01). Similarly, the data changes of the G2 group proved to be significant (p = 0.001) across the same time interval. In the comparison between the groups, VLQ values were not found to be significantly different (p > 0.05) at any point (Fig. 1).

**Table 1:** Characteristics of the study populations and results of the EQ-5D and the IMBK.

	G1	G2
**Number of participants**	15	15
**Age (years, mean ± SD)**	55.3 ± 4.2	54.3 ± 3.8
**Number of pregnancies (mean ± SD)**	2.20 ± 1.08	2.47 ± 1.5
**Number of children (mean ± SD)**	2.07 ± 0.88	1.60 ± 0.73
**Racial group (n, %)**		
White	9 (60%)	9 (60%)
Mixed race	6 (40%)	5 (33.3%)
Black	0 (0%)	1 (6.6%)
**Civil status (n, %)**		
Married	10 (66,6%)	6 (40%)
Divorced	4 (26,6%)	4 (26.6%)
Widow	1 (6,6%)	5 (33.3%)
	**(mean ± SD)**	**(mean ± SD)**
EQ-5D ‒ Pre-treatment	20.8 ± 2.7	20.4 ± 2.6
EQ-5D ‒ 60 days post-treatment	23.0 ± 1.7	21.8 ± 2.2
p-value	**0.001**^a^	**0.09**^a^
General health level ‒ pre-treatment	77.0 ± 15.0	78.6 ± 15.9
General health level ‒ post-treatment	71.6 ± 17.4	83,0 ± 9.9
p-value	**0.69**	**0.03**^a^
IMBK ‒ Pre-treatment	24.8 ± 7.8	27.7 ± 9.1
IMBK ‒ 60 days post-treatment	22.4 ± 7.0	24.7 ± 7.8
p-value	**0.007**^a^	0.05*

EQ-5D, EuroQol Five-Dimensional; IMBK, Blatt and Kupperman Menopausal Index; SD, Standard Deviation.

a Significant results.

The values obtained using the adapted EQ-5D questionnaire and the BKMI in the pre-treatment period, as well as 60 days after the initial session, are presented in Table 1. Statistical analysis revealed a significant difference in the values of the EQ-5D questionnaire in the G1 group (p = 0.001) only. Notably, based on the nominal distribution in this study, the authors found that a greater number of volunteers reported an ‘excellent’ score. The G2 group had a p-value of 0.09 for this questionnaire; however, an average difference of 11.4 points was observed for the general health level of this group, with a significance value of 0.03. There were no significant differences found in the comparison between the groups.

Concerning the BKMI, the intra-group comparison demonstrated a slight reduction in the means of the postmenopausal symptoms observed in both groups after the intervention. This was found to be significant for both G1 (p = 0.007) and G2 (p = 0.05). However, no significant differences were observed in a comparison between the groups.

### Photographic analysis

First, the photographs taken before the intervention were compared with those taken 30 and 60 days after the beginning of the protocol, which allowed us to observe the physical changes of the volunteers in each group.

**Fig. 2:**
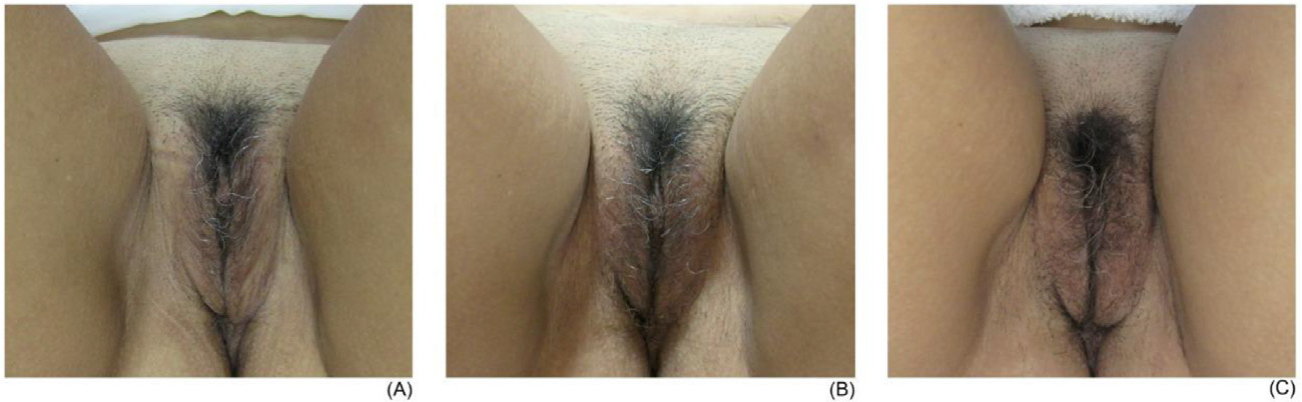
Example of a participant from the fractionated radiofrequency group: (A) initial assessment; (B) evaluation after 30 days; (C) evaluation after 60 days.

**Fig. 3:**
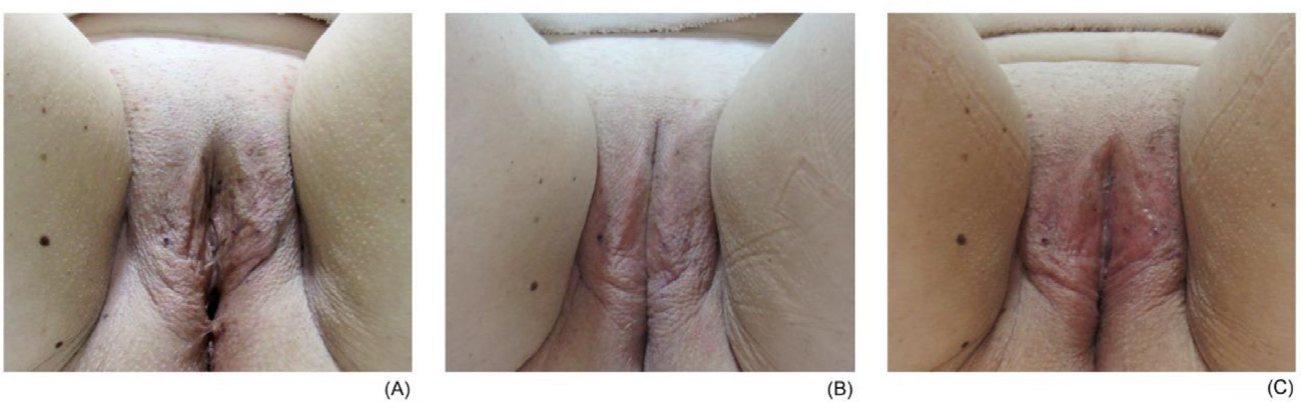
Example of a participant from the microneedling group: (A) initial evaluation; (B) evaluation after 30 days; (C) evaluation after 60 days.

In the initial photographs, the participants presented their vulva via a flaccid aspect, which was perceived via ripples in the tissue and areas with little filling. After a 30-day interval after the initial session, improvements in the quality of skin were observed. The region became more voluminous, with less wrinkled and flabby areas, with the skin color appearing more ‘reddish’. Thirty days after the second session, either maintenance or an improvement of the results were observed, mainly with a gain in volume and improvements in the color of the skin (Fig. 2, Fig. 3 and Fig. 2, Fig. 3).

**Fig. 4:**
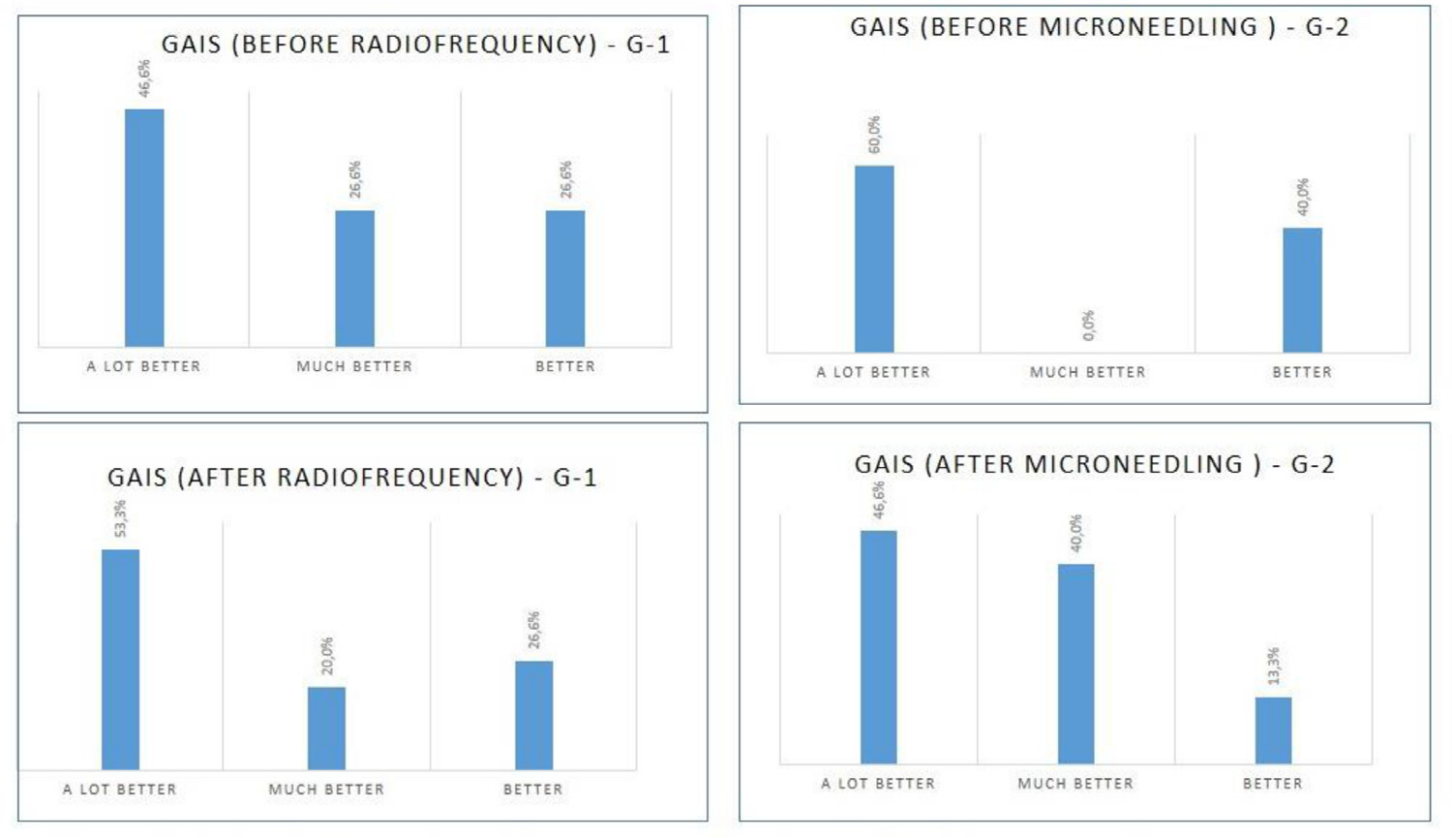
Results of the global aesthetic improvement scale: (A) G1 before undertaking an analysis of the photos; (B) G1 after analyzing the photos; (C) G2 before undertaking an analysis of the photos; (D) G2 after analyzing the photos.

During the application of the study therapies, most of the volunteers found it difficult to observe their intimate regions and analyze the changes after the interventions. Therefore, the authors decided to apply the GAIS at two different periods, as shown in Fig. 4.

The analysis of the scale revealed that the volunteers from both groups noticed positive differences in their vulvar region after the two treatment sessions, with the self-reports including the following responses: ‘A lot better’, ‘Much better’, and ‘Better’. For the G1 group, an increase in the number of respondents who rated their results as ‘A lot better’ was observed. As for the G2 group, there was an increase in the ‘Much better’ response; however, it must be noted that this change was made both by volunteers who observed better results in their photos and by those who had decided to reduce their classification.

Only three women from G1 (FRF) reported mild discomfort during the procedure. However, in none of the cases was the therapy stopped by the participants. All women resumed their daily activities, including sexual ones, immediately after each treatment session.

**Fig. 5:**
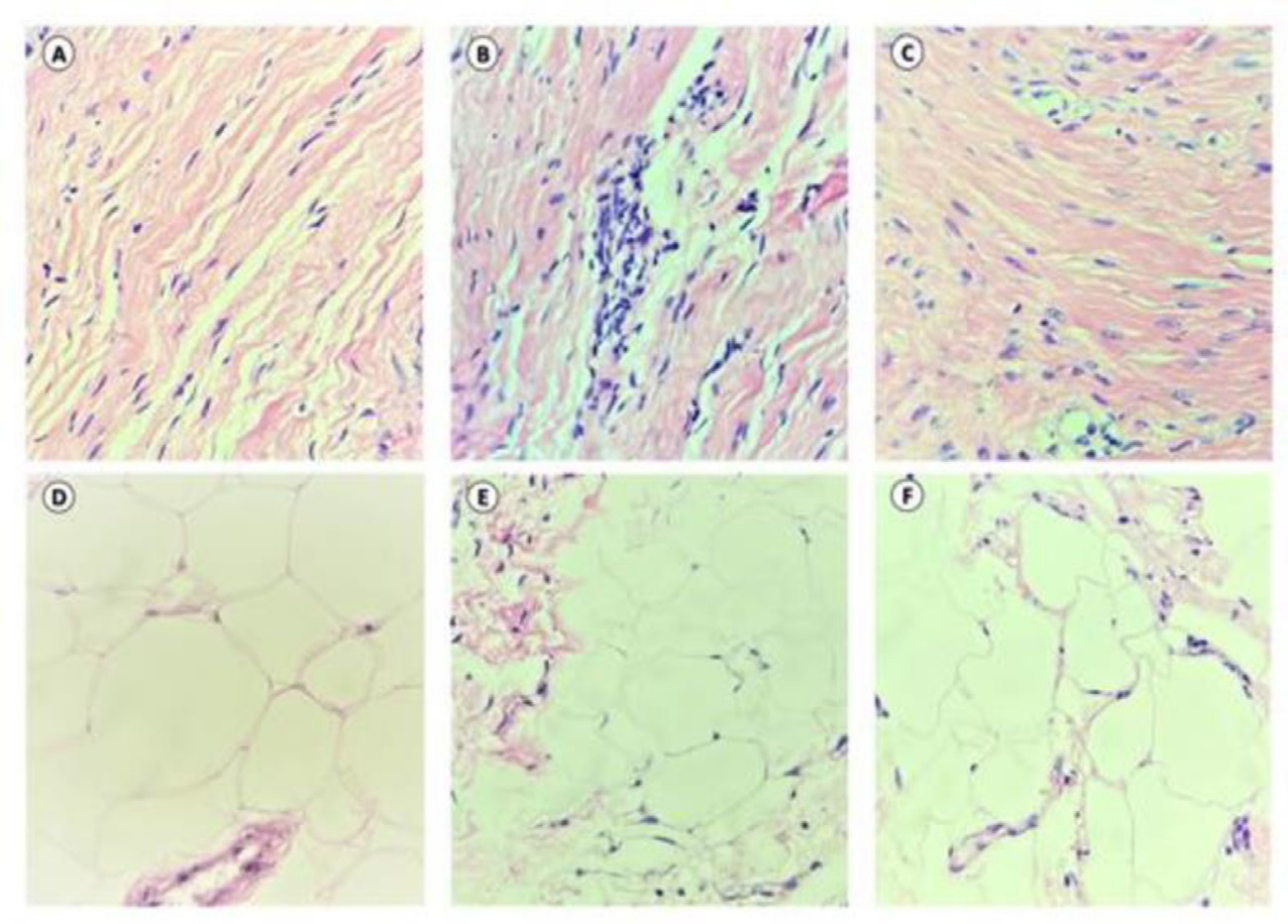
Morphological aspects of the skin treated with radiofrequency and Microneedling.

**Fig. 6:**
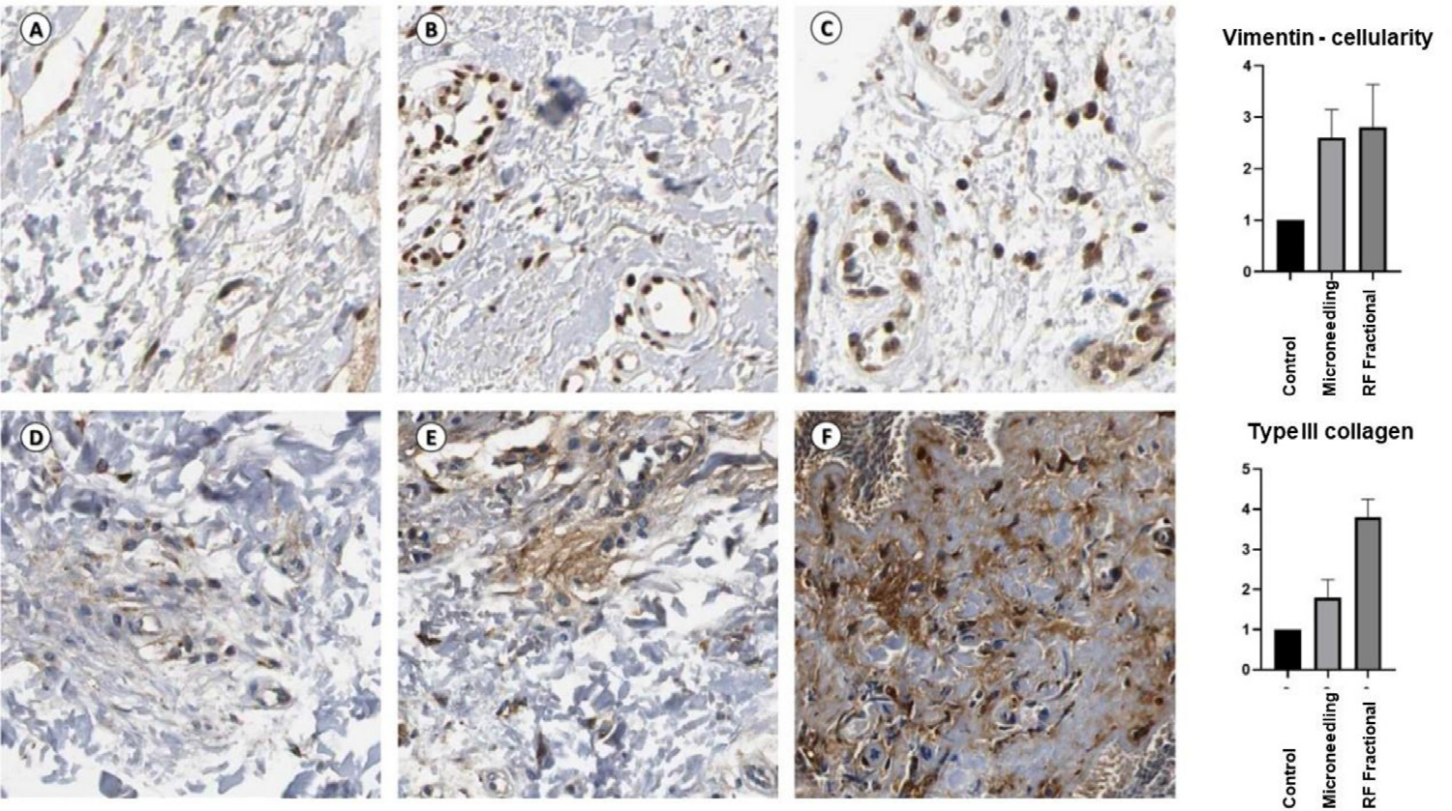
Immunohistochemical analyses of radiofrequency of the samples treated with radiofrequency and microneedling.

Regarding histological analysis, FRF demonstrated improvement concerning the number of fibroblasts, blood vessels, and fatty degeneration (p < 0.05) compared to the control (Fig. 5). During immunohistochemical analysis, FRF and microneedling samples showed higher type III collagen and vimentin expression in the (p < 0.05) (Fig. 6).

## Discussion

Due to the hormonal shortage that occurs at menopause, women have genitourinary atrophy and flaccidity. This sagging stems from the lack of collagen, and as a result, there are repercussions on urinary incontinence and genital prolapse.

Vulvar flaccidity improvements were observed in both study groups after two treatment sessions. However, there was no superiority between the techniques. These results are reinforced by previous studies that demonstrated that the FRF application system promotes the contraction of existing collagen fibers and stimulates the formation of new threads, thereby making them more efficient in supporting the skin.^21^^,^^25^ The latter technique allows for effective regeneration with low cost and a low rate of patient complications.^26^

The effect of microneedling is based that the resulting physical trauma, as provoked by needles, interacts with the papillary and reticular dermis in a purely mechanical way, leading to an induction of the body's standard response to wounding healing, causing minimal damage to the epidermis. The procedure then stimulates the deposition of fibroblasts and the formation and reorientation of collagen bundles, resulting in continuous tissue remodeling for months after the procedure.^27^

The efficiency of conventional RF devices, including those used on internal genitalia, has already been studied. A study using an RF device on the mucosal surface of the vaginal introitus of 30 premenopausal women with self-reported vaginal laxity after giving birth showed significant and sustained improvements in their laxity, sexual function, and sexual distress for as long as 12 months of follow-up.^28^ Fistonic et al.^29^ used a focused monopolar RF device to treat 17 women with labial laxity. They performed this treatment over four sessions, with intervals of seven days, with the patients then being followed up for one month after the last treatment. The results showed an improvement in the appearance of the vulva and the reported sexual gratification. The latter study reported a positive effect as a non-invasive treatment method for vulvar sagging despite limitations, including its small sample size, lack of a control group, and short follow-up period.

A previous study using RF involved 19 women aged between 35 and 64 years, who received four weekly treatment sessions lasting approximately 20 min each, demonstrated an improvement in their sexual function based on the Female Sexual Function Index (FSFI), with a statistically significant increase in five of the six FSFI dimensions. Additionally, these results were maintained after 12 months.^30^

Concerning the global aesthetic improvement scale, all volunteers related a positive result after undergoing the treatment about the However, the authors observed some difficulties faced by the patients regarding this evaluation. For example, individuals were able to easily perceive the aesthetic results on their face and body but, when these changes occurred in their more intimate regions, they weren't identified, either due to shame or a lack of habit of observing this area.^31^^,^^32^

The evaluation of the effectiveness of RF devices for genitourinary conditions is mainly based on subjective patient-reported outcomes. In this study, additionally, the authors performed histological and immunohistochemical analyses. Concerning histological analysis, FRF increased fibroblasts and blood vessels, besides fatty degeneration. FRF and microneedling samples showed higher type III collagen and vimentin expression during the immunohistochemical examination. Previous studies observed that heating tissues to temperatures less than 50 °C activate micro-remodeling through heat shock protein cascades, stimulating fibroblasts and initiating neocollagenesis and elastogenesis.^33^^,^^34^ In a study of the histological effects of a monopolar RF device in women with self-described mild to moderate vulvovaginal laxity, microscopic analysis was performed after three treatments targeting temperatures of 42°–45 °C. After completion of the three treatments, 80% of the samples from the vulva and 100% of those from the vagina demonstrated thickened mucosa and neovascularization. Neocollagenesis was noted on all post-treatment vulvar and vaginal samples.^35^^,^^36^

Limitations of this study include the relatively small number of participants enrolled. However, the present study is one of the few that describe the histological and immunohistochemical effects of RF and microneedling on genital tissue. The strength is that in previously published studies, the efficacy of RF on genitourinary was evaluated by subjective patient-reported outcomes. In addition, the participants in this study will be further followed up for one year, and the latter results will be reported in the future.

## Conclusion

Despite few studies on this topic, RF and microneedling seem to be options for all skin types, as they transfer less heat, causing less inflammation, and therefore, possess a lower risk of secondary hyperpigmentation. The present findings suggest that RF and microneedling were effective in treating the flaccidity of the female external genitalia, presenting histological changes suggesting collagen remodeling.

## Authors' contributions

Meyer PF, Maia RR, Sarmento AC, Carreiro EM, Farias SLQ, and Gonçalves AK were responsible for Conceptualization. Maia RR, Meyer PF, and Dantas CS were accountable for Methodology, and Maia RR was responsible for Project administration. The authors were responsible for the project resources. The Software Research Randomizer was used for the allocation sequence. Gonçalves, AK, performed the supervision. Soares CD did the validation, and Meyer PF and Gonçalves AK performed the visualization. The Writing – original draft, and the Writing – review & editing. Were performed by Gonçalves AK and Sarmento AC.

## Conflicts of interest

The authors declare no conflicts of interest.
